# Predictors of timely diagnostic follow-up after an abnormal Pap test among Hispanic women seeking care in El Paso, Texas

**DOI:** 10.1186/s12905-020-01161-9

**Published:** 2021-01-06

**Authors:** Thelma Carrillo, Jane R. Montealegre, Christina G. Bracamontes, Michael E. Scheurer, Michele Follen, Zuber D. Mulla

**Affiliations:** 1 Department of Obstetrics and Gynecology, Paul L. Foster School of Medicine, Texas Tech University Health Sciences Center El Paso, El Paso, TX USA; 2 Department of Pediatrics, Baylor College of Medicine, Houston, TX USA; 3 Dan L. Duncan Comprehensive Cancer Center, Baylor College of Medicine, Houston, TX USA; 4 Present Address: NYC Health + Hospitals| Kings County, Brooklyn, NY USA; 5 Office of Faculty Development (MSC 21007), Texas Tech University Health Sciences Center El Paso, 5001 El Paso Drive, El Paso, TX 79905 USA

**Keywords:** Inequity, Colposcopy, Adherence, Cervical cancer, Hispanic, Texas-mexico border

## Abstract

**Background:**

Diagnostic follow-up of women with an abnormal Pap test is necessary to resolve the risk developing cervical cancer. The purpose of this study is to describe patient characteristics associated with timely receipt of a diagnostic colposcopy after an abnormal Pap test among Hispanic women in El Paso, a Texas-Mexico border city.

**Methods:**

We conducted a retrospective chart review of Hispanic patients seen at an academic colposcopy clinic following an abnormal Pap test. An optimal diagnostic interval to colposcopy was based on a National Breast and Cervical Cancer Early Detection Program (NBCCEDP) quality indicator and was defined as receipt of colposcopy within 90 days or less from the date of an abnormal Pap test. Risk ratios (RR) were calculated by building a generalized linear model fit using a Poisson distribution, log link, and robust variance.

**Results:**

Overall, 177 of the 270 women (65.6%) received follow-up within an optimal diagnostic interval. After adjusting for other variables in the model, women who were 30 years of age or older were 32% more likely to have an optimal interval than younger women (adjusted RR = 1.32, *P* < 0.01). High school graduates were less likely than more educated women to have an optimal interval (adjusted RR = 0.68, *P* < 0.01). Participation in the NBCCEDP was not associated with receipt of follow-up within an optimal diagnostic interval.

**Conclusions:**

Compared with women with greater educational attainment, high school graduates were less likely to receive follow-up within an optimal diagnostic interval, as were younger (≤ 30 years) women compared with older women. Participation in the NBCCEDP was not associated with receipt of care within an optimal diagnostic interval.

## Background

Cervical cancer incidence and mortality have decreased dramatically since widespread screening with the Papanicolaou test became available in the 1940s [1]. However, in 2015 there were an estimated 12,900 new cases of invasive cervical cancer and 4,100 deaths in the United States (U.S.) [2]. Cervical cancer incidence and mortality are highest among minority women, specifically among African-American and Hispanic women [3], with respective incidence and mortality rates of 9.0 and 4.0 per 100,000 among African Americans and 9.5 and 2.5 per 100,000 among Hispanic women compared with 7.1 and 2.1 per 100,000 among non-Hispanic Whites [2, 4, 5]. In 2013, Hispanic women in Texas had the second highest cervical cancer mortality rates among Hispanic women in the nation, with mortality rates of 3.1 per 100,000 [5]. The cervical cancer mortality rates among women along the Texas-Mexico border are reported to be even higher than for women living in non-border counties in Texas. In the years 2002–2006, Hispanic women along the Texas-Mexico border had a cervical cancer mortality rate of 4.6 per 100,000 compared with 2.5 per 100,000 in non-Hispanic White women living in non-border counties [6]. Unsurprisingly, women along the border are less likely to report a recent Pap test compared with women in non-border counties in Texas, 61.7% vs. 75.7% [7] and are substantially less likely to have health insurance, 52.6% vs. 73.7% [7].

While racial/ethnic disparities in cervical cancer incidence are primarily due to decreased access and utilization of screening, lower rates of diagnostic and therapeutic follow-up among Hispanic and African American women also contribute to higher cervical cancer mortality among these groups [8–10]. Recent studies suggest that 13 to 40% of cervical cancer diagnoses result from lack of follow-up among women with an abnormal screening test [11–13]. Diagnostic follow-up typically involves a colposcopy with biopsy of areas with apparent abnormalities [14] and treatment recommendations are based on these results. While there are no data to clearly define a specific interval in which receipt of diagnostic follow-up optimally increases survival, longer time to treatment has been shown to result in later stage disease and, consequently, poorer survival [15].

Barriers to timely diagnostic follow-up are complex and include inadequate access to healthcare services, communication barriers between patients and providers, inexperience navigating the health care system, and psychological distress [16–18]. The National Breast and Cervical Cancer Early Detection Program (NBCCEDP), which provides low-income, uninsured, and underserved women access to timely breast and cervical cancer screening and diagnostic services, aims to address these barriers through evidence-based interventions at multiple levels of the social ecological model [19–21].

In this analysis, we examine the characteristics of predominantly low-income Hispanic women living along the Texas-Mexico border who received timely follow-up diagnostic care after receiving their abnormal Pap smear results. Specifically, we examined characteristics of women who obtained their colposcopy exam within the 90 day follow-up interval established by the NBCCEDP as a quality indicator. Given that Hispanic women along the U.S-Mexico border are among the demographic groups with the highest cervical cancer incidence and mortality rates in the U.S., identifying characteristics associated receiving timely follow-up is crucial for the development of interventions to deliver timely care to this population.

## Methods

### Participants

This was a retrospective cohort study of women presenting for diagnostic colposcopy at an academic healthcare center (Texas Tech University Health Sciences Center, TTUHSC) in El Paso, Texas between 2012 and 2014 following their receipt of an abnormal Pap test result. TTUHSC receives NBCCEDP funding distributed from the state of Texas and since 2007 has provided care to over 2,700 El Paso residents enrolled in the NBCCEDP program. A woman who is between 21 and 64 years of age who has an intact uterus and does not have health insurance or whose health insurance carrier does not cover Pap testing is eligible for free cervical cancer screening via the NBCCEDP if her family income is ≤ 250% of the federal poverty level [21]. Some states, however, use a different poverty threshold namely ≤ 200% of the federal poverty level [21].

Participants in this study were those taking part in a larger National Institutes of Health-sponsored clinical trial to evaluate emerging optical technologies for diagnosis of cervical dysplasia [22, 23]. The protocol of the parent study (Development and Application of a Multispectral Digital Colposcope and Probe Algorithm for Detection of Cervical Intraepithelial Neoplasia) was reviewed and approved by the TTUHSC Institutional Review Board (IRB) for the Protection of Human Subjects (IRB # E12117). A written informed consent was obtained from each of the study subjects. The current project was a quality assurance/quality improvement investigation and hence did not require IRB approval. Of the 329 participants enrolled in the trial, 13 were excluded because they were not of Hispanic ethnicity or had an unknown Hispanic ethnicity status. Another 46 were excluded because they had missing values for one or more of the variables that were included in the multivariate analysis. These exclusions resulted in a final sample size of 270 participants.

### Procedure

Medical records of trial participants were reviewed to ascertain their cervical cytology history, particularly the date of their last Pap test, the result of their last Pap test, and the date that they attended the colposcopy exam. A questionnaire regarding sociodemographic and risk factor characteristics was administered at the clinic by trained, bilingual/bicultural interviewers prior to the procedure.

### Measures

An optimal interval between an abnormal Pap test and colposcopy was defined as receiving a colposcopy within ≤ 90 days of an abnormal Pap test result [20]. Subjects whose interval between the date of their abnormal Pap smear and the date of their colposcopy was 90 days or less were classified as having an optimal interval. Subjects whose interval was greater than 90 days were classified as not having an optimal interval.

Sociodemographic characteristics included age (≥ 30 years versus < 30 years), education less than high school, high school graduate, greater than high school, place of birth (U.S. vs. other country), participation in the NBCCEDP, and acculturation. In regards to educational attainment, subjects were asked, “What is the highest level of education you have completed?”.

Acculturation was measured by the Marín Short Acculturation Scale for Hispanics (SASH) [24]. Study subjects answered four questions concerning what language(s) they read, speak, and think in. The response for each of the four questions ranged from 1 to 5 with the following coding scheme: 1 = Only Spanish, 2 = Spanish better than English, 3 = Both Equally, 4 = English better than Spanish, 5 = Only English. The responses provided by the subjects was averaged across the four items. Subjects whose mean SASH score was greater than 2.99 were classified as “more acculturated” while those with a mean SASH score of 2.99 or less were classified as “less acculturated.” Hispanic ethnicity was assessed with the question, “Are you of Hispanic, Latino or Spanish origin?” and categorized as No-Not of Hispanic Latino or Spanish origin, Yes-Mexican-Mexican American-Chicano, Yes-Puerto Rican, Yes-Cuban, Yes-another Hispanic-Latino or Spanish origin, Don't Know, or Refused.

Smoking status was used as a surrogate measure of risk-taking behaviors given the findings of previous investigators [25, 26]. For example, Escobedo et al. [26] studied a nationally representative sample of adolescents from the United States and found that cigarette smoking was correlated with having multiple sexual partners among Hispanic females. Participants in our study were classified as smokers if they had smoked 100 or more cigarettes during their entire life. Clinical characteristics included the result of the last Pap test results, which was dichotomized as high-grade result versus normal or low-grade result.

### Statistical analysis

Data were analyzed using SAS 9.3 software (SAS Institute, Inc., Cary, North Carolina). The outcome of interest was an optimal diagnostic interval. Frequencies and Pearson’s chi-square tests were used to compare demographic, epidemiologic, and clinical characteristics among participants who had an optimal diagnostic interval and those who did not. The following variables (detailed above) were deemed to be of clinical and/or epidemiological importance and hence were entered in our regression models: age, educational level, Hispanic acculturation status, place of birth, participation in the NBCCEDP, smoking status, and a high-grade result on the last Pap test.

Our initial attempt at a multivariable analysis involved fitting a log-binomial regression model using the GENMOD Procedure and specifying a binomial distribution with a log link; however, this model did not converge. As an alternative, Poisson regression with a robust variance was used to examine factors that were associated with having an optimal diagnostic interval [27]. Collinearity among the predictor variables was not detected using a logistic regression model. Adjusted risk ratios (RR) from the multiple Poisson regression model were reported along with 95% confidence intervals (CI) and p-values. Associations with a p-value ≤ 0.05 were considered statistically significant.

## Results

A total of 270 Hispanic women were included in the analysis. The majority of these women (98.5%) identified themselves as being of Mexican, Mexican–American, or Chicano origin while the remaining 1.5% were Puerto Rican or of another Hispanic, Latino, or Spanish origin. Additional characteristics of the sample are reported in Table 1. Approximately 66% of the subjects had an optimal interval (≤ 90 days) between the date of their abnormal Pap smear and the date the colposcopy was performed. The median number of days from the abnormal Pap to colposcopy was 59 days (range 9 to 90 days) among women who received care in the optimal interval and 148 days (range 91–516 days) among those who received follow-up care after 90 days. A total of 13 women had both a high-grade lesion and a sub-optimal interval (> 90 days), and none of these women were diagnosed with cancer (data not shown). Of the 177 subjects who had an optimal interval, 60.5% were ≥ 30 years of age, and of the 93 subjects who did not have an optimal interval, 40.0% were ≥ 30 years of age (*P* = 0.001). The prevalence of being more acculturated (versus less acculturated) was 41.2% in the optimal interval group and 57.0% in the sub-optimal group (*P* = 0.01). Women who had an optimal interval were less likely to be born in the United States than subjects who did not have an optimal interval: 46.3% vs. 59.1% (*P* = 0.045).

**Table 1:** Characteristics of 270 Hispanic women with an abnormal Pap smear, categorized by the duration of their diagnostic interval

Characteristic	Had an optimal interval^a^ N = 177 Number (%)	Did not have an optimal interval^a^ N = 93 Number (%)	*P*
*Demographic and epidemiologic*			
Age ≥ 30 years	107 (60.5)	37 (40.0)	0.001
Educational level			0.17
Less than high school	48 (27.1)	21 (22.6)	
High school graduate	32 (18.1)	26 (28.0)	
Greater than high school	97 (54.8)	46 (49.5)	
More acculturated (vs. less)^b^	73 (41.2)	53 (57.0)	0.01
Place of birth			0.045
United States	82 (46.3)	55 (59.1)	
Outside of the United States	95 (53.7)	38 (40.9)	
Participant in the National Breast and Cervical Cancer Early Detection Program	88 (49.7)	50 (53.8)	0.53
Smoked ≥ 100 cigarettes in entire life	39 (22.0)	27 (29.0)	0.20
*Clinical and clinico-pathologic*			
High-grade result on Pap smear	33 (18.6)	13 (14.0)	0.33

^a^Defined as 90 days or less

^b^As measured by the Marín Short Acculturation Scale for Hispanics (SASH)

Adjusted RRs are presented in Table 2. After adjusting for other variables in the model, having an optimal interval to colposcopy was associated with age and education. Women who were 30 years of age or older were 32% more likely to have an optimal interval than younger women. High school graduates were 32% less likely than those with a higher education to have an optimal interval (RR = 0.68, 95% CI: 0.53–0.88). The association between acculturation and having an optimal interval that was detected in the univariate analysis (Table 1) was not observed after adjusting for the remaining six predictor variables. Participation in the NBCCEDP program was not associated with having an optimal interval.

**Table 2:** Adjusted^a^ risk ratios (RR) for an optimal interval (≤ 90 days) between the date of an abnormal Pap smear and date of colposcopy in 270 Hispanic women

Variable	RR	95% confidence interval	*P*
*Demographic and epidemiologic*			
Age ≥ 30 years (vs. age < 30 years)	1.32	1.09–1.61	0.005
Educational level			
Less than high school	0.84	0.67–1.04	0.11
High school graduate	0.68	0.53–0.88	0.004
Greater than high school	1	(Referent)	–
More acculturated (vs. less acculturated)	0.82	0.65–1.02	0.08
Place of birth			
United States	0.93	0.75–1.15	0.51
Outside of the United States	1	(Referent)	–
Participant in the National Breast and Cervical Cancer Early Detection Program (vs. not a participant)	1.03	0.87–1.23	0.74
Smoked ≥ 100 cigarettes in entire life (vs. < 100)	0.86	0.69–1.07	0.19
*Clinical and clinico-pathologic*			
High-grade result on Pap smear (vs. other result)	1.13	0.92–1.39	0.25

RRs are from a Poisson regression model with robust variance estimation

^a^Each RR is adjusted for the remaining variables found in the table

## Discussion

One of the major factors contributing to cervical cancer incidence in our and other medically underserved populations is non-receipt or untimely receipt of diagnostic and therapeutic follow-up after an abnormal Pap test [8–10]. In our sample, about 66% of patients had an optimal diagnostic interval as defined by the NBCCEDP. In El Paso, NBCCEDP patients receive patient navigation, a service that has been shown to improve timely follow-up among medically underserved women [28]. However, among cases in our analysis, NBCCEDP status was not associated with receipt of care within an optimal diagnostic interval. The reason for this null association requires further investigation. We feel that multiple factors could impact the length of the diagnostic interval independent of funding status including the reluctance of patients to undergo colposcopy, lack of transportation [29], variations in provider practice patterns, and issues surrounding the scheduling of appointments.

A brief discussion of the cost of a colposcopy is merited at this point. While our NBCCEDP-funded patients were not charged for undergoing colposcopy, patients who paid cash (self-pay patients) for a colposcopy were billed USD 369 by our institution during fiscal year 2014. Self-pay patients were charged USD 454 for a colposcopy with biopsy (biopsies) during fiscal year 2014.

A recent national-level analysis of NBCCEDP data indicated that in 2009, 71% of NBCCEDP patients met a 60-day diagnostic interval, the performance measure at the time, with a larger proportion of the programs (> 80%) meeting the current 90-day diagnostic interval during the period 2003–2009 [20]. However, the same study found racial and ethnic disparities in diagnostic intervals, with the longest wait times for diagnosis after a screening-detected abnormality among black and Hispanic women [20]. The median diagnostic intervals in that study were 47 days in White women, 48 days in Asian women, and 50 days in both Black and Hispanic women [20]. The barriers that limit timely diagnostic resolution among Hispanic NBCCEDP participants nationwide are likely similar to those experienced in El Paso and further study is needed to elucidate them.

Being born outside the U.S. was also hypothesized to be associated with a longer interval to diagnostic colposcopy given that immigrants often face barriers to healthcare access, including lack of insurance, lack of awareness of available resources, and linguistic and cultural barriers [29]. However, while US-born women in our sample were more likely than foreign-born women to exceed an optimal diagnostic interval, this association did not remain statistically significant after controlling for age and other variables.

We did find a significant association between both age and education and attendance to colposcopy within an optimal diagnostic interval. Women who were ≥ 30 years of age were 32% more likely than younger women to have an optimal interval between the time of their abnormal Pap smear and the performance of the colposcopy. This finding is consistent with other studies that have also reported a higher likelihood to default from follow-up diagnostic care among younger women [12, 30]. Younger women are significantly less likely than older women to have health insurance coverage, leaving them vulnerable to high out-of-pocket expenses for medical procedures such as colposcopy [31]. Women under 30 years may also face increased distress barriers compared with older women [32], particularly related to potential reproductive consequences such as the loss of fertility which may be a consequence of the treatment for cervical cancer [33, 34].

In regard to education, we found that timely attendance to colposcopy was more likely among Hispanic women with more than a high school education compared with those with a high school education only. Interestingly, the likelihood of timely attendance to colposcopy was similar among women with less than a high school education compared with those with a post-high school education. While Sharp et al. reported that women with post-high school education were more likely to attend for colposcopy [35], education in their study was dichotomized as having post-high school education versus not. Our results suggest that in populations with a high proportion of individuals with less than a high school education, such as border Hispanic women, the association between education and attendance for diagnostic follow-up may be more nuanced.

The findings of this study should be interpreted within the context of its limitations. Because women were recruited at a colposcopy clinic, our study sample only includes women who ultimately attended for colposcopy. Inclusion of non-attenders in future studies may better elucidate the role that patient characteristics play in predicting timely follow-up. We did not assess the role of other individual-level barriers such as knowledge about cervical cancer prevention, language barriers impeding understanding and communication with health care providers, inexperience in navigating the health care system, and lack of health insurance. Similarly, we were unable to elucidate social and cultural impediments for the receipt of diagnostic care, such as fear of stigmatization and psychological fears, including *fatalismo*, or fatalistic fears which may impede individuals from seeking care [36, 37]. Despite these limitations, our results provide valuable data describing timeliness of cervical cancer diagnosis among a unique group of medically underserved Hispanic women. Clinicians should be aware of increased likelihood of non-attendance among younger women and those with a high school education only. Patient education efforts can focus on this group to reinforce the need for clinical follow-up. In 2016, 16.6% of Texans did not have health insurance coverage [38]. In contrast, the prevalence of individuals without health insurance in the U.S. in 2016 was 8.6% [38]. Given the high proportion of persons living without health insurance in Texas, the NBCCEDP continues to be a relevant and important initiative.

Finally, research is needed to determine how grantees of NBCCEDP funds can better ensure timely screening, diagnostic and treatment care among eligible Hispanic women. Marlow and colleagues conducted a qualitative study (using six focus groups) to investigate barriers to cervical cancer screening among women 50 to 64 years of age in England [39]. Those authors identified several reasons why some women did not attend cervical cancer screening including embarrassment, discomfort, and a lack of an invitation letter (some participants had not received prompts to be screened from a healthcare professional) [39]. Holding focus groups of women of various ages and conducting key informant interviews of community health workers and health care providers in our region may aid us in identifying interventions that will decrease the interval between an abnormal Pap smear result and colposcopy.

## Data Availability

The dataset for the current study is available from the corresponding author upon receipt of a reasonable request.

## Abbreviations

CI: Confidence interval
NBCCEDP: National Breast and Cervical Cancer Early Detection Program
RR: Risk ratio
SASH: Short Acculturation Scale for Hispanics
US: United States
