# Correction to: Predictors of timely diagnostic follow-up after an abnormal Pap test among Hispanic women seeking care in El Paso, Texas

**DOI:** 10.1186/s12905-021-01186-8

**Published:** 2021-01-27

**Authors:** Thelma Carrillo, Jane R. Montealegre, Christina G. Bracamontes, Michael E. Scheurer, Michele Follen, Zuber D. Mulla

**Affiliations:** 1 Department of Obstetrics and Gynecology, Paul L. Foster School of Medicine, Texas Tech University Health Sciences Center El Paso, El Paso, TX USA; 2 Department of Pediatrics, Baylor College of Medicine, Houston, TX USA; 3 Dan L. Duncan Comprehensive Cancer Center, Baylor College of Medicine, Houston, TX USA; 4 Present Address: NYC Health + Hospitals| Kings County, Brooklyn, NY USA; 5 Office of Faculty Development (MSC 21007, Texas Tech University Health Sciences Center El Paso, 5001 El Paso Drive, El Paso, TX 79905 USA

## Correction to: BMC Women’s Health (2021) 21:11 10.1186/s12905-020-01161-9

Following publication of the original article [1], we were notified of a typographical error in the Conclusions section of the Abstract: “ ≤ 30 years” should actually read “ < 30 years”.

The original article has been corrected.
